# An intrapleural administration of zoledronic acid for inoperable malignant mesothelioma patients: a phase I clinical study protocol

**DOI:** 10.1186/s40064-016-1893-2

**Published:** 2016-02-27

**Authors:** Yuji Tada, Kenzo Hiroshima, Hideaki Shimada, Masato Shingyoji, Toshio Suzuki, Hiroki Umezawa, Ikuo Sekine, Yuichi Takiguchi, Koichiro Tatsumi, Masatoshi Tagawa

**Affiliations:** Department of Respirology, Graduate School of Medicine, Chiba University, Chiba, Japan; Department of Pathology, Tokyo Women’s Medical University Yachiyo Medical Center, Yachiyo, Japan; Department of Surgery, School of Medicine, Toho University, Tokyo, Japan; Division of Respirology, Chiba Cancer Center, Chiba, Japan; Department of Medical Oncology, Faculty of Medicine, University of Tsukuba, Tsukuba, Japan; Department of Medical Oncology, Graduate School of Medicine, Chiba University, Chiba, Japan; Division of Pathology and Cell Therapy, Chiba Cancer Center Research Institute, 666-2 Nitona, Chuo-ku, Chiba, 260-8717 Japan; Department of Molecular Biology and Oncology, Graduate School of Medicine, Chiba University, Chiba, Japan

**Keywords:** Mesothelioma, Bisphosphonates, Clinical study, Chemotherapy, Pleural cavity

## Abstract

**Background:**

The third generation of bisphosphonates is clinically in use for patients of osteoporosis or malignancy-linked hypercalcemia. The agents can also produce anti-tumor effects on bone metastasis of several types of tumors. We recently found that one of the agents achieved cytotoxicity to mesothelioma in vitro and in an orthotopic animal model. Mesothelioma is resistant to a number of chemotherapeutic agents, and suppression of local tumor growth is beneficial to the patients since metastasis to extra-thoracic organs is relatively infrequent until a late stage.

**Methods/design:**

We demonstrated in an orthotopic mouse model that an intrapleural but not intravenous injection of zoledronic acid, one of the third generation bisphosphonates, at a clinically equivalent dose suppressed the tumor growth. Nevertheless, a high concentration of zoledronic acid administrated in the pleural cavity produced pleural adhesion. We also showed that zoledronic acid produced synergistic cytotoxic effects with cisplatin, the first-line chemotherapeutic agent for mesothelioma. We then planned to conduct a phase I clinical study to investigate any adverse effects and a possible clinical benefits produced by an intrapleural administration of zoledronic acid to mesothelioma patients who became resistant to the first-line chemotherapeutic agents. The clinical trial is a dose escalation study starting with 0.4, 1, 4, 8 and 16 mg per person since safety of administration of zoledronic acid into the pleural cavity remains unknown. Each dose group consists of three persons and the protocol allows to repeat administration of the same dose into the pleural cavity at a 4-weeks interval.

**Discussion:**

We will conduct a possible combinatory study of intrapleural administration of zoledronic acid and systemic administration of the first-line agent to a chemotherapy-naïve patient based on the maximum tolerance dose of zoledronic acid determined by the present clinical trial. We propose that administration of bisphosphonates in a closed cavity is a treatment strategy for tumors developed in the cavity probably through the direct cytotoxic activity.

*Trial registration:* UMIN clinical trials registry, Japan. Register ID: UMIN8093.

## Background

Malignant mesothelioma is often associated with occupational usage of asbestos in the patient’s history but some cases are also linked with non-occupational exposures (Carbone et al. 2002; Robinson et al. 2005; Porpodis et al. 2013; Røe and Stella 2015). A majority of mesothelioma develop from pleura and malignant pleural mesothelioma invades along the cavities even in an early phase. The invasiveness consequently suppresses functions of vital organs in the pleural cavity, resulting in respiratory and cardiac failure. Nevertheless, distant metastasis to extrathoracic organs is relatively infrequent until the late stage, indicating that suppression of the local tumor growth can be beneficial to the patients.

Mesothelioma is resistant to current multimodal treatments (Opitz 2014; Kotova et al. 2015). Extrapleural pneumonectomy is one of the standard surgical options for an early-staged case, but recurrence is often common. Moreover, quality of the patient life deteriorates due to the extensive operation procedure. Mesothelioma is not suitable for radiotherapy because a high dose radiation is required and a wide radiation area causes pneumonitis. It is subsequently used for a palliative purpose. Chemotherapy is currently the primary treatment in most of the cases, and a combination of cisplatin (CDDP) and pemetrexed (PEM) is the first-line chemotherapeutic regimen. Nevertheless, the mean survival period with the combination is 12.1 months (Vogelzang et al. 2003) and no second-line regimen has yet been established for more than 10 years. Many clinical trials with different anti-cancer agents including molecular target agents did not show any improvement in the survival (Tada et al. 2015). A new therapeutic agent is therefore required to improve the prognosis.

Detection of mesothelioma at an early stage is often difficult, and differential diagnosis from other cancerous and non-cancerous diseases needs careful pathological examinations including an immunohistochemical staining with several kinds of antibody. The latent period after asbestos exposure is long, more than 30 years in an average, and a medical procedure to prevent the tumor development is currently unavailable. Many industrial countries have terminated to use asbestos, but emerging countries especially in Asia rather increase asbestos consumption due to their economic development (Lin et al. 2007).

**Fig. 1:**
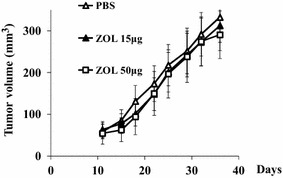
Growth of subcutaneous tumors developed in BALB/c mice. MSTO-211H cells (3 × 10^6^) were subcutaneously inoculated and the mice were injected with PBS or ZOL (15 or 50 μg) in the intraperitoneal cavity twice a week after day 11. Tumor volume was calculated according to the formula (1/2 × length × width^2^). The tumor volumes were not statistically different at any time in any groups. The average and SE *bars* are shown (n = 6)

We recently demonstrated that the third generation of bisphosphonates, zoledronic acid (ZOL), achieved anti-tumor effects on mesothelioma (Okamoto et al. 2012). The agent induced cell cycle arrest and apoptosis in mesothelioma through inhibiting functions of small G proteins and the activity of topoisomerase II (Okamoto et al. 2014). ZOL treatments also augmented p53 expression levels in mesothelioma bearing the wild-type p53 gene and the increased p53 expression contributed to synergistic combinatory cytotoxicity by ZOL and CDDP (Okamoto et al. 2013). These data suggested that ZOL was one of the potential candidates for mesothelioma treatments. ZOL is currently used for osteoporosis and for malignancy-induced hypercalcemia in a clinical setting. The agent tends to accumulate in bone tissues and blocks activities of osteoclasts, resulting in lowering serum calcium concentrations (Yuasa et al. 2007). Previous studies demonstrated that ZOL also suppressed metastasis of breast and prostate cancer to bone tissues (Climent et al. 2013; Mathew and Brufsky 2015). We then examined a possible anti-tumor effects of ZOL in vivo and found that a systemic administration of ZOL failed to achieve anti-tumor effects on subcutaneous mesothelioma (Fig. 1). In contrast, an intrapleural injection of ZOL suppressed mesothelioma developed in the pleural cavity (Okamoto et al. 2012). Furthermore, an administration of ZOL in the pleural cavity produced combinatory anti-tumor effects with CDDP in the orthotopic animal model (Okamoto et al. 2013). These preclinical studies suggested that an intrapleural administration achieved a high concentration of ZOL enough to produce cytotoxic effects, which prompted us to conduct a clinical trial to examine safety and possible clinical benefits of ZOL administered into the pleural cavity. We therefore planed a phase I study at Chiba University Hospital, Chiba, Japan, for inoperable and chemotherapy-failed mesothelioma patients. We showed our clinical protocol and some of preclinical data regarding the safety of ZOL injected into an intrapleural cavity.

### Efficacy of intrapleural administration of ZOL for mesothelioma

We examined possible anti-tumor effects of intrapleural injection of ZOL in an orthotopic animal model, human mesothelioma tumors inoculated in the thoracic cavity of BALB/c nude mice (Table 1). All the animal experiments described were approved by the animal experiment and welfare committee at Chiba Cancer Center Research Institute and Chiba University. Administration of ZOL into the intrapleural cavity suppressed the tumor developments with a dose-dependent manner irrespective of tumor cells inoculated. Body weights of mice injected with MSTO-211H cells were greater in the ZOL-treated group than in control group. The differential body weights changes seemed to reflect extension of tumors and subsequent emaciation processes. A decreased body weight gain is one of the indicators to estimate possible toxicity and these data indicated that ZOL administration did not cause serious adverse reactions. Growth of MSTO-211H cells in vitro was faster than that of EHMES-10 cells, which was attributable to greater tumor weights of MSTO-211H cells than EHMES-10 cells.

**Table 1:** Inhibited tumor growth and body/heart weights after an intrapleural injection of ZOL

Agent (μg/mouse)	Day of agent administration	Tumor weight (average ± SE) (mg)	Body weight (average ± SE) (g)	Heart weight (average ± SE) (mg)
Experiment 1 (assessment of tumor weight: day 28)				
PBS	3	491.1 ± 68.4**	ND	ND
ZOL (80 μg)	3	0**	ND	ND
Experiment 2 (assessment of tumor weight: day 28)				
PBS	3	384.8 ± 36.8**	15.9 ± 5.1**	99.8 ± 5.2*
ZOL (40 μg)	3	19.8 ± 7.2**	18.9 ± 0.24**	114.5 ± 3.6*
Experiment 3 (assessment of tumor weight: day 35)				
PBS	3	197.7 ± 22.9**	18.5 ± 1.0	123.2 ± 5.8
ZOL (40 μg)	3	2.5 ± 1.2**	19.6 ± 0.3	129.5 ± 3.5*
ZOL (15 μg)	3	79.3 ± 13.2**	19.2 ± 0.2	116.6 ± 3.4*
Experiment 4 (assessment of tumor weight: day 28)				
PBS	10	377.9 ± 13.3*	16.1 ± 0.4*	ND
ZOL (40 μg)	10	188.6 ± 62.1*	19.2 ± 0.5*	ND

BALB/c nude mice were inoculated with human mesothelioma, MSTO-211H cells (experiment 1, 2 and 4) or EHMES-10 (experiment 3) (1 × 10^6^/mouse), in the intrapleural cavity and were injected with PBS or ZOL (100 μl in volume) on day 3 or 10. Tumor weights were measured on the indicated day and the averages with standard errors are shown (experiment 1; n = 5, experiment 2; n = 6, experiment 3; n = 7, experiment 4; n = 7)

** P < 0.01; * P < 0.05 (experiment 3; difference of tumor weights is statistically significant in any of two groups and that of heart weights is statistically significant only between ZOL 15 μg- and ZOL 40 μg-injected groups)

We also calculated a mouse dose equivalent to the human dose that was clinically in use. Provided that an average body weight of a human and a mouse are 60 kg and 20 mg, respectively, 4 mg of ZOL, the human clinical dose, corresponds to 16,4 μg in mouse with a conversion equation (Reagan-Shaw et al. 2008). We observed suppression of tumor growth at 15 μg of ZOL in an orthotopic animal model with EHMES-10 cells (Table 1). These data suggested that 4 mg of ZOL suppressed growth of mesothelioma in the pleural cavity. The plasma concentrations at 6 and 24 h after an intravenous administration of 4 mg ZOL were about 10 and 2 ng/ml, respectively (open data from Novartis, ZOMU00007). Supposing that 4 mg ZOL is injected into the intrapleural cavity of a patient with 1000 ml pleural effusion, the concentration in the pleural cavity is 4 μg/ml. The concentration was significantly higher than the plasma concentration when the same amount of ZOL was used. Our previous cytotoxicity data in vitro showed that ZOL at more than 3 μg/ml in the concentration achieved growth suppression of all the human mesothelioma cells tested (Okamoto et al. 2012). These data collectively indicated that the current clinical dose of ZOL, when injected in the pleural cavity of an effusion-positive case, could produce anti-tumor effects.

Another possible mechanism of ZOL in the anti-tumor effects in vivo is associated with activated immune responses. ZOL inhibits farnesyl pyrophosphate synthase in the mevalonate pathway, and subsequently increases concentrations of isopentenyl pyrophosphate and triphosphoric acid I-adenosine-50-yl ester 3-(3-methylbut-3-enyl) ester (ApppI), an ATP analogue, in the treated tumor cells. These molecules contain a binding motif of T cell receptor complexes of γδ Τcells (Wada et al. 2014). Consequently, γδ Τcells bearing the Vγ9 Vδ2 T cell receptors were activated by ZOL-treated cells (Benzaid et al. 2011) and kill the ZOL-treated tumors with interferon (IFN)-γ secretion (Märten et al. 2007). Nevertheless, a precise mechanism how the activated human γδ Τcells produce cytotoxicity to the tumors remains uncharacterized.

### Preclinical study for safety in intrapleural administration

Administration of ZOL in the pleural cavity has not been examined in human being. A high concentration of ZOL may produce adverse reactions since ZOL can induce inflammatory responses by producing proinflammatory cytokines (Scheller et al. 2011). A clinical study was conducted for gastric cancer patients with malignant ascites with intraperitoneal injection of ZOL at 1 mg for priming cytotoxic γδ Τcells (Wada et al. 2014). The study showed that the ZOL administered into the peritoneal cavity did not clinically produce any adverse effects but induced recruitment of neutrophil in the peritoneal cavity. Norton et al. (2012) also reported that alendronate, an US Food and Drug Administration-approved bisphosphonate, induced inflammatory reactions in the peritoneal cavity in mice. We thereby investigated possible adverse effects induced by intrapleural injections of ZOL with BALB/c nude mice. Administration of 80 μg ZOL in 100 μl of phosphate-buffered saline (PBS) produced adhesion between pleura and pericardium in a majority of the mice tested, but 40 μg of ZOL did not induce the adhesion. Heart weights also increased in mice injected with ZOL at 40 μg (experiment 2) on day 28 compared with those with PBS, but the mild cardiomegaly was not detected on day 35 (experiment 3). An average heart weight of mice that received ZOL at 15 μg was even lower than that of mice injected with PBS. The mechanism of weight gain of heart in mice is unknown but it may be associated with a possible congestive heart failure listed as one of the serious adverse effects of ZOL in human.

**Fig. 2:**
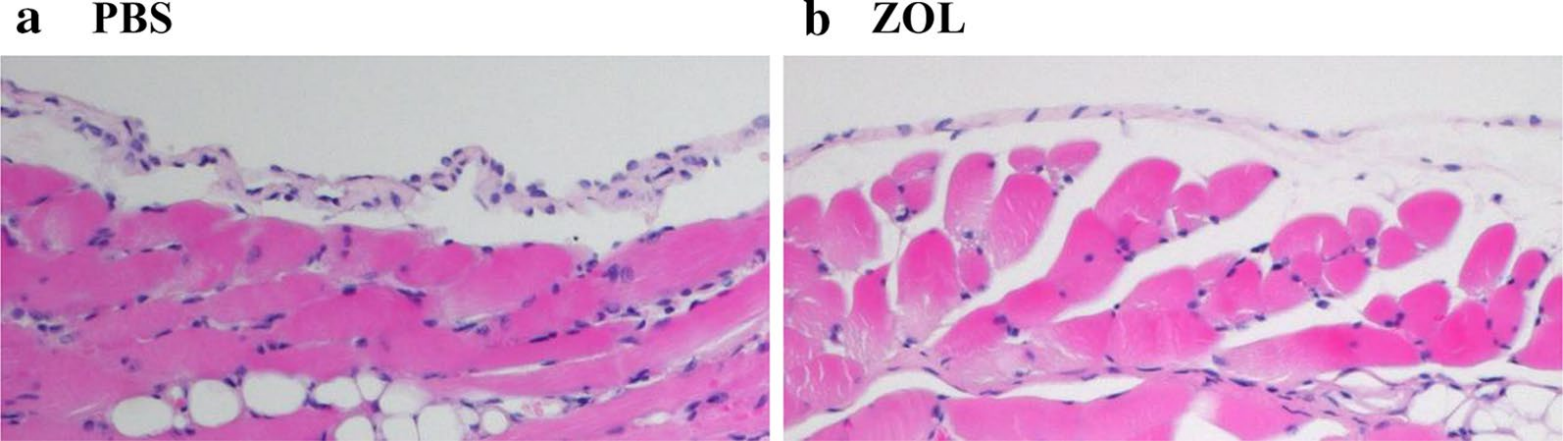
Histological pictures of parietal mesothelium of BALB/c nude mice injected with **a** PBS or **b** ZOL (40 μg) on day 25. Hematoxylin and eosin staining with ×20 magnifications

We also pathologically examined pleura of mice injected with ZOL or PBS as a control (Fig. 2). Treatments with ZOL at 40 μg in 100 μl of PBS did not show any cellular infiltrations in pleura, adipose tissues and muscles beneath the pleura. We did not find inflammatory reactions and furthermore, the histological features were not different from those of PBS-injected mice. These data indicated that ZOL less than 0.4 μg/μl at the concentration was safely used in mice.

Administration of anti-cancer agents into the pleural cavity are relatively uncommon but previous studies showed that an injection of the chemotherapeutic agent into the pleural cavity could be conducted safe (Jones et al. 2010; Baba et al. 2013). Constant lung movements also facilitate contact and spread of an agent to mesothelioma. Nevertheless, it is unclear whether administration into the intrapleural cavity maintains a relative high concentration of an agent and induces less frequent systemic toxicity compared with an intravenous administration in malignant pleural diseases. A drug concentration in the pleural cavity is influenced not only by an amount of pleural effusion but also by pleural pathophysiological conditions that were controlled by invasion levels of malignancy. Clinical studies with ZOL for hypercalcemia associated with malignancy, multiple myeloma or metastatic bone lesion, showed that a majority of ZOL-induced adverse effects was fever, general malaise, hypocalcemia and hypokalemia. The serious adverse effects include acute renal failure, congestive heart failure and osteonecrosis of the jaw, but the frequency of these serious reactions was less than 1 % of the cases. The planned a phase I study nevertheless needs to pay attention to the above adverse effect as for safety.

## Study design and objectives

**Fig. 3:**
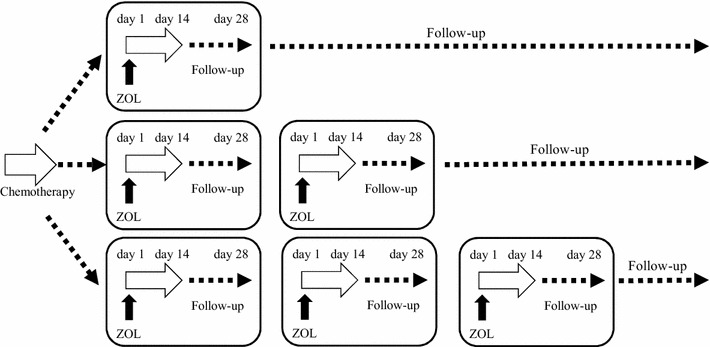
A schematic design of the clinical study. The examination for safety ends 14 days after the ZOL injection but the patient are followed up for the efficacy until day 28 and every 4 weeks thereafter. The patient can repeatedly receive ZOL in the pleural cavity at a 4-weeks interval

The phase I study design is to administer ZOL in 100 ml saline solution into the pleural cavity of mesothelioma patients who are not suitable for a surgical operation and fail to respond to the first-line chemotherapeutic agents (Fig. 3). It is a dose escalation study with ZOL at 0.4, 1, 4, 8, 12, 16 mg per person in a single injection and three patients are included in each dose group. The protocol allows to repeatedly use the same ZOL dose for a 4-weeks interval when the patient wants to receive it and is compatible with the inclusion criterial described below. The observation period in the current protocol is 14 days as for the safety, but we also follow up them until day 28 for the efficacy and continue to see them at a 4-weeks internal thereafter in our outpatient clinic. Escalation to the next dose is dependent on the frequency of adverse effects. Three more patients need to be enrolled when 1 patient in a group has an adverse effect greater than the grade 3 level of the common terminology criteria for adverse events (CTCAE) ver 4.0, and the clinical study must to be terminated when more than two patients out of total six cases have adverse effects in the same dose group.

The primary endpoints are to investigate a safety level of ZOL injected into the pleural cavity, which includes to define the maximum tolerance dose and to clarify any adverse effects produced according to CTCAE ver 4.0. The secondary endpoints are evaluation of anti-tumor effects based on tumor volumes, amounts of pleural effusion estimated with radiological imaging, and any improvements of the patients’ quality of life and the performance status. The tumor volume is assessed with the modified Response Evaluation Criteria in Solid Tumors (Byrne and Nowak 2004; Labby et al. 2012), and the subjective pain condition is measured with a visual analogue scale. Evaluation of the anti-tumor effects will be conducted on day 14 and also on day 28 when the patient does not receive any treatments. The clinical protocol was approved by the institutional review board of the Chiba University Hospital (G23070) and that of Graduate School of Medicine, Chiba University (TIBADAI-IN No: 205), and was registered at UMIN clinical trials registry, Japan (UMIN 8093).

### Subject selection and withdrawal

#### Inclusion criteria

Patients eligible for the study are those who are pathologically diagnosed as malignant pleural mesothelioma of locally advanced or recurrent cases, and not suitable for surgical resection. Those refuse to undergo the surgery irrespective of their clinical stages are also eligible. In addition, the patients who become refractory to chemotherapy or decline to receive chemotherapeutic agents are eligible. Patients, aged between 20 and 80 years old, must be fully explained about the study using a document and give a written informed consent. Patients must have pleural effusion to have an enough space in the pleural cavity where ZOL is injected. The Eastern Co-operative Oncology Performance status must be between 0 and 2. Life expectancy of the patients will be longer than 3 months. Patients need to have adequate physiological functions in major organs and the laboratory findings must be as follows: white blood cell; ≧3000/mm^3^ or neutrophil; ≧1500/mm^3^, platelet; ≧1 × 10^5^/mm^3^, hemoglobin; ≧8.0/dl, total bilirubin; ≦1.5 mg/dl, aspartate transaminase and alanine aminotransferase; ≦100 IU/l, creatinine; ≦1.5 mg/dl, creatinine clearance; ≧60 ml/min; partial pressure of oxygen in arterial blood; ≧60 Torr or SPO_2_ (breathing in room air) ≧90 %, electric cardiogram; within normal range.

The principle investigators and the medial collaborators including those responsible for monitoring the current study must approve that the patient is in an adequate condition to be enrolled in the study.

#### Exclusion criteria

Patients with a different tumor other than mesothelioma, either synchronous or metachronous, are not eligible unless they are completely cured or their progression free interval is longer than 2 years. Patients who have symptomatic brain metastatic foci or those who require a treatment for the brain metastasis are excluded. Patients who do not have an enough intra-thoracic space for ZOL injection or have participated in other clinical trial(s) with approved or unapproved medicine within 4 weeks before the entry of this study are not allowed to be enrolled. Those who are scheduled to receive another anti-cancer drug(s) during the study period and those who received pleurodesis or radiation within 1 month as a mesothelioma treatment are excluded. Patients who have interstitial pulmonary diseases and pulmonary fibrosis judged by chest computed tomographic scanning are not eligible. Patients who had myocardial infraction before 6 months at the entry and still have a risk of possible recurrence, who have experienced allergic reactions or adverse effects greater than the grade 3 level of CTCAE ver 4.0 following ZOL or other bisphosphonates administrations, and who have dental diseases that require invasive treatments are not eligible.

Those who have some factors that can prevent good compliance with the study protocol and the follow-up schedules, including some psychiatric, psychological, familial, social or geographical issues, are regarded as not being suitable for the study. Patients who are judged as inappropriate to participate in the study by the principal investigators and the collaborators are excluded.

## Discussion

The study is planned to investigate possible adverse effects and clinical benefits produced by an intrapleural injection of ZOL to malignant pleural mesothelioma patients. The bisphosphonates have not yet been administered into the pleural cavity of human being and our preclinical study indicated that a high concentration of ZOL induced adhesion in pleura and pericardium probably due to some inflammatory reactions. An intravenous administration of ZOL produced benefits to several types of cancer patients with bone metastasis although controversial data about the efficacy were also reported (Zekri et al. 2014). The mechanism underlying the possible anti-tumor effects was linked with accumulation of ZOL in bone tissues since the effects were not produced against tumors developed in or metastasized to non-osseous sites. In contrast, the present clinical protocol aims to examine the direct cytotoxicity of ZOL at a high concentration in non-osseous tissues.

A previous study reported that ZOL plasma concentrations rapidly declined with half-lives of 0.2 h (Chen et al. 2002). The maximum plasma concentration achieved by an intravenous injection of 4 mg ZOL did not reach to a concentration that was required to kill mesothelioma cell lines in vitro. In contrast, an intrapleural administration in an orthotopic mouse model can achieve the concentration necessary to stop cell proliferation in vitro and in fact we demonstrated the anti-tumor effects in vivo. A clinical application of the intrapleural administration needs to consider two factors, possible inflammatory reactions that may be associated with adverse reactions and maintenance of a ZOL concentration in the pleural cavity. An intrapleural concentration of ZOL is crucial for the adverse effects as well as growth inhibitory activity, but the concentration is difficult to estimate because of difficulty to assess a volume of pleural effusion. Moreover, we cannot determine tissue distributions of ZOL since an enzyme-linked immunosorbent assay for ZOL is currently unavailable. Continuously monitoring of ZOL concentrations in the pleural cavity is not clinically practical. The current phase I trial is thereby a dose escalation study to investigate possible adverse reactions according to CTCAE ver 4.0 and compared with those in the case of an intravenous administration. Several serious adverse effects such as acute renal failure, congestive heart failure and osteonecrosis of the jaw reported in the intravenous injection will be also carefully monitored in this clinical trial. In clinical settings, an intravenous injection of ZOL induces some of inflammatory reactions including fever, general malaise and flu-like syndrome. Bisphosphonates in fact can activate non-specific immune responses accompanied by neutrophil chemotaxis and relevant cytokine productions (Scheller et al. 2011; Norton et al. 2012; Wada et al. 2014), which may be linked with pleural and pericardial adhesion observed in our preclinical study. Wada et al. (2014) conducted a clinical trial for patients with peritoneal dissemination by administering Vγ9 Vδ2 T cells and 1 mg of ZOL into the peritoneal cavity to activate the γδ T cells. They demonstrated that a ZOL concentration in ascites fluid was greater and sustained for longer period in the intraperitoneal cavity than in the intravenous injection. The study also showed that granulocytes were recruited in the peritoneal cavity and the patients had low-grade fever. It is currently unknown whether these non-specific inflammation reactions were due to ZOL administration since the γδ T cells were administered at the same time and IFN-γ was also produced by the activated γδ T cells.

Cytotoxicity of ZOL against mesothelioma is ascribable to several mechanisms including inhibition of small G proteins and topoisomerase II activity (Okamoto et al. 2012, 2014). The ZOL-induced cell death was associated with apoptotic processes but not linked with autophagy, and many factors were involved in the cell death processes. An ATP analogue of ApppI which was produced by a feedback mechanism, mediated by ZOL-induced depletion of isoprenoid and subsequent enhancement of the mevalonate pathway, could play a role in the apoptosis, and ZOL-mediated Rab inhibition also contributed to the cell death process (Okamoto et al. 2014). Disrupted actin fiber structures after ZOL treatments were caused by suppressed Rho functions and consequently induced cell detachment followed by cell death (Okamoto et al. 2014). Moreover, mesothelioma treated with ZOL showed augmented p53 expression levels and the p53 phosphorylation when the p53 genotype was wild-type (Okamoto et al. 2012). The augmentation further increased sensitivity to CDDP, the first-line agent for mesothelioma. These data collectively indicate that ZOL produces anti-tumor responses to mesothelioma when they are directly contacted with the agent in vivo.

Mesothelioma is histologically classified into three subtypes, epithelioid, biphasic and sarcomatoid types. Sensitivity to chemotherapy is different among the subtypes and prognosis of sarcomatoid type is in general the worst because of the resistance to chemotherapeutic agents. A recent study interestingly suggested a polyclonal origin of mesothelioma, which may reflect the differential chemosensitivity among the types (Comertpay et al. 2014). Consequently, the prognosis depends on a ratio of heterogeneity within tumors. A clinical response to ZOL can be linked with the histological subtypes and is differentially influenced by the dependency on small G proteins for the cell growth and on production of the cytotoxic ATP analogue. The present study however does not take the histological classification into consideration because of limited numbers of the enrolled patients. Moreover, our previous cytotoxic study in vitro showed that ZOL sensitivity was not correlated with histological types of the cell lines (Okamoto et al. 2012). Stimulation of immune responses through the γδ T cells with the specific T cell receptors is another interesting point for investigation. The present study however will not investigate the activation of the γδ T cells since detection of the T cells in peripheral blood cells and regional lymph nodes needs careful examinations, which include a time-course study and exclusion of non-specific T cell activations by inflammatory cytokines.

In conclusions, the present study plan is to evaluate safety and efficacy of ZOL administered in the pleural cavity. Confirmation of the safety will lead us to possible subsequent clinical studies, which can be a combinatory use of ZOL and the first-line chemotherapy to a chemotherapy-naïve patient, and a phase II study with the same protocol using the maximum tolerance dose of ZOL.

## Abbreviations

ApppI: triphosphoric acid I-adenosine-50-yl ester 3-(3-methylbut-3-enyl) ester
CDDP: cisplatin
CTCAE: common terminology criteria for adverse events
IFN: interferon
PBS: phosphate-buffered saline
PEM: pemetrexed
ZOL: zoledronic acid
